# Intermolecular And Dynamic Investigation of The Mechanism of Action of Reldesemtiv on Fast Skeletal Muscle Troponin Complex Toward the Treatment of Impaired Muscle Function

**DOI:** 10.1007/s10930-023-10091-y

**Published:** 2023-03-23

**Authors:** Abdul Rashid Issahaku, Mahmoud A. A. Ibrahim, Namutula Mukelabai, Mahmoud E. S. Soliman

**Affiliations:** 1 Molecular Bio-Computation and Drug Design Laboratory, School of Health Sciences, University of KwaZulu-Natal, Westville Campus, Durban, 4001 South Africa; 2 CompChem Research Group, Chemistry Department, Faculty of Science, Minia University, Minia, 61519 Egypt; 3 Department of Physiotherapy, School of Health Sciences, University of KwaZulu- Natal, Westville Campus, Durban, 4001 South Africa; 4 West African Centre for Computational Research and Innovation, Accra, Ghana

**Keywords:** Fast skeletal troponin, Troponin C, Troponin I, Molecular dynamics simulation, Reldesemtiv

## Abstract

Muscle weakness as a secondary feature of attenuated neuronal input often leads to disability and sometimes death in patients with neurogenic neuromuscular diseases. These impaired muscle function has been observed in several diseases including amyotrophic lateral sclerosis, Charcot–Marie–Tooth, spinal muscular atrophy and Myasthenia gravis. This has spurred the search for small molecules which could activate fast skeletal muscle troponin complex as a means to increase muscle strength. Discovered small molecules have however been punctuated by off-target and side effects leading to the development of the second-generation small molecule, Reldesemtiv. In this study, we investigated the impact of Reldesemtiv binding to the fast skeletal troponin complex and the molecular determinants that condition the therapeutic prowess of Redesemtiv through computational techniques. It was revealed that Reldesemtiv binding possibly potentiates troponin C compacting characterized by reduced exposure to solvent molecules which could favor the slow release of calcium ions and the resultant sensitization of the subunit to calcium. These conformational changes were underscored by conventional and carbon hydrogen bonds, pi-alkyl, pi-sulfur and halogen interactions between Reldesemtiv the binding site residues. Arg113 (−3.96 kcal/mol), Met116 (−2.23 kcal/mol), Val114 (−1.28 kcal/mol) and Met121 (−0.63 kcal/mol) of the switch region of the inhibitory subunit were among the residues that contributed the most to the total free binding energy of Reldesemtiv highlighting their importance. These findings present useful insights which could lay the foundation for the development of fast skeletal muscle small molecule activators with high specificity and potency.

## Introduction

Skeletal muscles are responsible for most bodily movements. Upon receipt of a minute electric signal from the brain via neurons, skeletal muscles rapidly contract though the simultaneous contraction of several muscle fibers which result in driving joint and skeletal movements [1]. In several diseases, muscle fatigue and weakness are a product of limited neural input and often lead to substantial disability and increased mortality [2]. Neuropathies such as amyotrophic lateral sclerosis, Charcot–Marie–Tooth and spinal muscular atrophy result in motor neuron damage and death affecting the ability of healthy motor neurons to stimulate muscles effectively to generate force [3–6]. As seen in myasthenia gravis, muscle weakness and fatigue are caused by the failure of signal transmission at the neuromuscular junction which result in limiting calcium release and force production [7]. Myasthenia gravis treatment therefore involves acetylcholinesterase inhibitors and immunosuppression though weakness and fatigue are still observed in treated individuals [8].

**Fig. 1:**
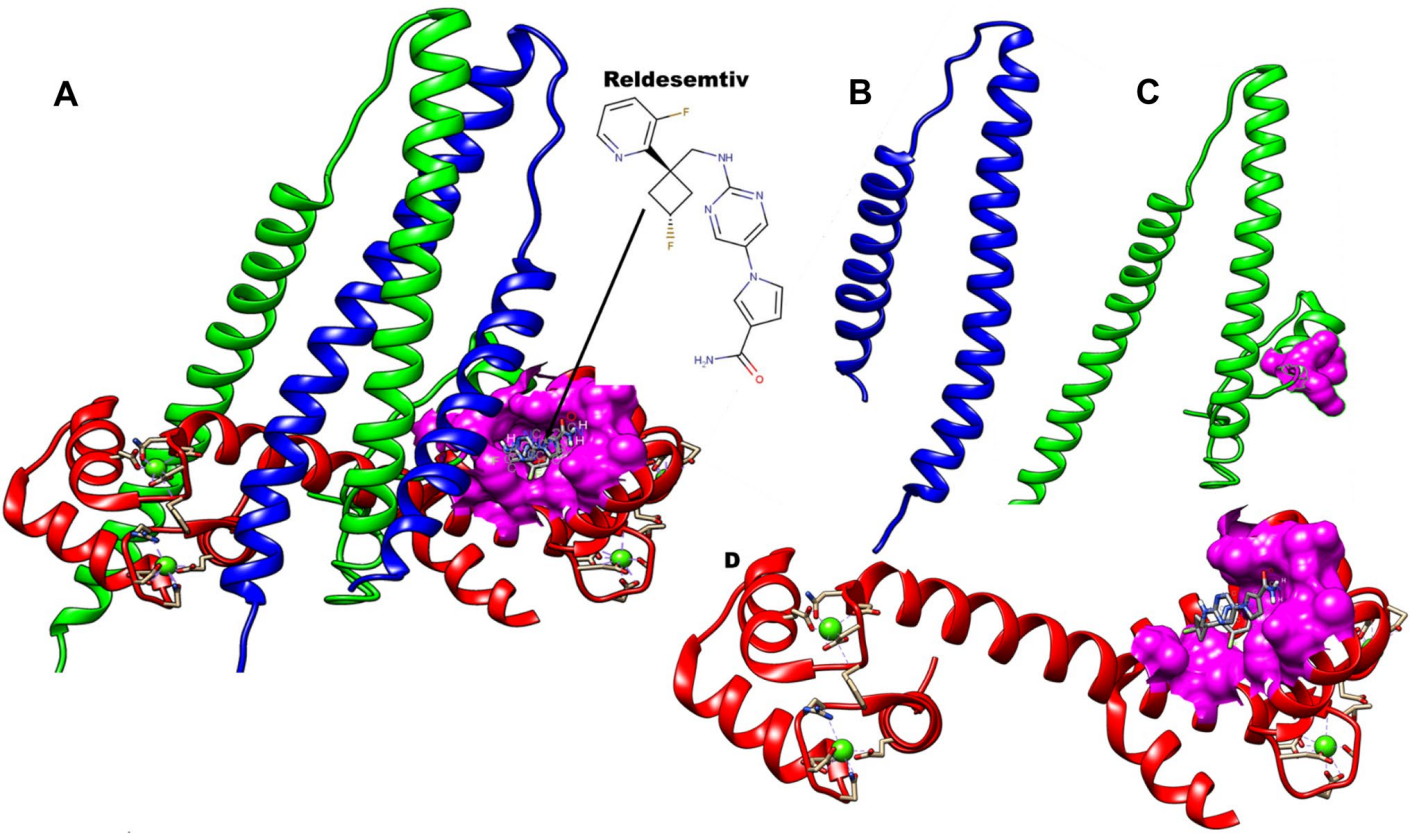
Structure of fast skeletal muscle troponin complex (PDB ID; 1YTZ). **A** shows the complex highlighting the binding pocket of small molecule activators (magenta) with Reldesemtiv docked inside. **B** shows the fast skeletal troponin T (sTnT) subunit (blue ribbons). **C** shows the fast skeletal troponin I (sTnI) subunit (green), magenta region shows the area that partakes in interactions with Reldesemtiv. **D** shows the fast skeletal troponin C (red) subunit with highlight on the binding site on the N-terminal (magenta). Green balls show calcium ions bound to the subunit (Color figure online)

Although edaravone and nusinersen have been approved recently for the treatment of amyotrophic lateral sclerosis (ALS) and spinal muscle atrophy (SMA), treatment options are limited and represent a significant unmet medical need [9–12]. Direct activation of skeletal muscle has therefore become a therapeutic target to improve the physical performance in patients with compromised muscle function [13]. In the process of muscle contraction, the muscle sarcomere shortens due to the sliding of the thick filaments built of myosin molecules along the thin filament [14]. Troponin, tropomyosin, and actin make up the thin filament [15, 16]. While actin bears the binding sites for the sub fragment 1 region of myosin, tropomyosin controls the availability of these sites and troponin controls the accessibility in a Ca^2+^ dependent manner [17]. Skeletal muscles are grouped into two main forms, Fast skeletal muscle (type I) and slow skeletal muscle (type II) which vary in their usage, metabolism and sarcomere composition [18]. Fast skeletal muscle fibers vary from cardiac and slow muscles in the uniqueness of its troponin and myosin components [19]. Myosin acts as the molecular motor that mediates contractility where chemical energy from adenosine triphosphate (ATP) hydrolysis is coupled to force generation while the troponin complex performs a regulatory role by acting as Ca^2+^ sensor [20]. Troponin comprises three subunits: Troponin T (TnT), Troponin I (TnI), and Troponin T (TnC) [21] as shown in Fig. 1. TnT binds to tropomyosin and positions the troponin complex on the thin filament. TnI interrupts myosin ATPase cycle in the presence of actin at low Ca^2+^ levels while TnC reacts to the increase in Ca^2+^ concentration by annulling TnI inhibition [22, 23]. Serving as the Ca^2+^ sensor, TnC contains four EF-hands [24] which opens upon the binding of Ca^2+^ with an accompanying exposure of a hydrophobic cavity [20, 25]. This cavity is exploited by TnI switch segment to potentiate other conformational changes to the thin filament, accelerating myosin binding [26]. Sequence homology between fast and slow muscle troponins C, T, and I is about 50% offering an opportunity to target fast skeletal muscle therapeutically [27]. Several efforts have therefore been channeled to the discovery of troponin activators. These efforts led to the discovery of the first direct troponin activator, Tirasemtiv through high throughput screening (HTS) and optimization [28]. Tirasemtiv selectively interacts with fast skeletal muscle troponin complex inducing an increase affinity for calcium and amplifies the muscle cell response to neural input hence increasing force production at submaximal muscle stimulation frequencies [13, 28, 29]. However, in phase 3 clinical trials for the treatment of ALS, Tirasemtiv did not produce the desired slow vital capacity [30] coupled with side effects from GABA receptor potentiation which caused dizziness [31, 32] leading to the discovery of the second generation small molecule Reldesemtiv [33, 34].

Reldesemtiv exhibits the same mechanism of action as Tirasemtiv and has shown to induce increased force production at submaximal stimulation frequency compared to Tirasemtiv in in vivo studies and phase 1 clinical trials and is therefore currently under clinical studies for the treatment of amyotrophic lateral sclerosis [34, 35]. This study sought to unravel the atomistic and molecular insights into the therapeutic activity of Reldesemtiv on the troponin complex through computational techniques and molecular dynamics simulation. It is our hope that insights unraveled herein will aid in the design of selective small molecule activators with improved therapeutic prowess and less side effects on fast skeletal muscle.

## Methodology

### System Preparation

The X-ray crystal structures of the troponin complex [20] with PDB ID 1YTZ and the structure of troponin C in complex with Tirasemtiv [36] (PDB ID 7KAA) were retrieved from the Protein Data Bank (PDB) [37]. Since Tirasemtiv and Reldesemtiv have the same mechanism of action, the structure 7KAA, was used in pre-analysis by superimposition with 1YTZ to identify the active site on the troponin complex. The structure of Reldesemtiv was drawn using Marvin Sketch 18.10.0 [38] and Avogadro 1.2.0 [39] employed to build the 3D structure and optimize it as well and then saved in mol2 format. The troponin complex was prepared using UCSF Chimera 1.13.1 [40] by deleting all non-standard residues and the protein saved in pdb format. Calcium ions which were co-crystalised with the structure and are reported to play a critical role in Troponin C function was removed because they did not conform to standard AMBER parameterization. The ions were however replaced with Amber standardized ions retrieved from the University of Manchester amber database [41, 42]. Gasteiger charges were then added and AutoDock Vina 4.2 [43] employed to dock the Reldesemtiv to the binding site of the complex. Tirasemtiv was redocked into the binding pocket and the binding poses compared to the co-crystallised pose. An RMSD of 0.61 Å was observed for the first generated pose. This assured us that AutoDock Vina can satisfactorily predict the correct binding pose for the structure. A gid box of centre X = 18.226, Y = 2.24258 and Z = −21.1203 with size X = 21.6265, Y = 18.27, and Z = 17.7503 was used to direct the docking process. The best pose with the least energy score (most negative) was selected and saved for the ensuing further preparations. With UCSF Chimera, AMBCC charges and hydrogens were added to the ligand before subjecting it and the receptor to parameterization. Two systems were created: the unbound troponin complex system with the labile calcium ions (apo) and the Reldesemtiv bound troponin (Ca^2+^) complex system.

### Molecular Dynamic Simulation

The bound and the unbound systems were subjected to molecular dynamics simulation using the Graphic Processing Unit (GPU) version of Particle Mesh Ewald Molecular Dynamics (PMEMD) of AMBER18 [44]. The systems were parameterized with the FF14SB force field [45]. Atomic partial charges for the ligand was generated and added using ANTECHAMBER [46] which utilized the Restrained Electrostatic Potential (RESP) and the general amber force field (GAFF) protocols [47]. Additional hydrogens were then added and the systems neutralized by adding Na^+^ and Cl^−^ counter ions and the systems solvated using the LEap module [48] incorporated in AMBER18. The systems were suspended in bulk solvent of Transferable Intermolecular Potential with 3 Points (TIP3P) water box size of 10 Å. Before MD simulation production, the systems were minimized and relaxed, heated and then equilibrated. 10,000 steps of minimization with the steepest descent were carried out and then switched to the conjugate gradient algorithm to further minimize the systems for 5000 steps with a restraint potential of 500 kcal/molA^2^. Heating was then performed gradually from 0 K to 300 k for 5 ps in isothermal-isobaric NTP ensemble using Langevin thermostat [49] with a pressure of 1 bar by employing Berendsen barostat [50]. The systems were then equilibrated at 300 k for 1000 ps without energy restraint. Afterwards, MD production was performed for 150 ns with the SHAKE algorithm [51] used to restrain all hydrogen bonds at 2 fs time step.

CPPTRAJ and PTRAJ modules [52] of AMBER18 were used to analyze all the trajectories and coordinates generated from the MD production. Origin data tool version 6.0 [53] was used to plot the graphs while visualization was performed with Discovery Studio v19.10.18289 [54] and UCSF Chimera.

### Thermodynamics Estimations

The Molecular Mechanics/Poisson-Boltzmann Area (MM/PBSA) method was applied for the investigation due to its efficiency and widely reported reliability [55]. The energies were averaged over 3,000 snapshots generated from the trajectories of the 150 ns simulation. The binding free energy of this approach is depicted as follows:

1 $${\Delta G}_{{{\text{bind}}}} {\text{ = G}}_{{{\text{complex}}}} {\text{{-}G}}_{{{\text{receptor}}}} {\text{{-}G}}_{{{\text{ligand}}}}$$

2 $${\Delta G}_{{{\text{bind}}}} {\text{ = E}}_{{{\text{gas}}}} {\text{ + G}}_{{{\text{sol}}}} {\text{{-}T}}\Delta {\text{S}}$$

where ΔG_bind_ is taken to be the sum of the gas phase and solvation energy terms less the entropy (TΔS) term

3 $${\text{E}}_{{{\text{gas}}}} {\text{ = E}}_{{{\text{int}}}} {\text{ + E}}_{{{\text{vdw}}}} {\text{ + E}}_{{{\text{ele}}}}$$

where E_gas_ is the total of the AMBER force field internal energy terms. E_int_ (bond, angle and torsion), the covalent van der Waals (E_vdw_) and the non-bonded electrostatic energy component (E_ele_). The solvation energy is denoted by the equation:

4 $${\text{G}}_{{{\text{sol}}}} {\text{ = G}}_{{{\text{PB}}}} {\text{ + G}}_{{\text{non - polar}}}$$

5 $${\text{G}}_{{\text{non - polar}}} {\text{ = SASA}}$$

The polar solvation contribution is denoted as G_PB_ and G_non-polar_ represents the non-polar contribution energy and is computed from the solvent assessable surface area (SASA). Which is obtained using 1.4A water probe radius. Per-residue decomposition analyses were also performed to estimate individual energy contribution of the residues of the substrate pocket to the affinity and stabilisation of the compounds.

## Results and Discussion

In many diseased conditions, limited neuronal input lead to muscle weakness due a decrease in the intense of muscle innervation, the frequency of neuromuscular junction activation or the effectiveness of synaptic transmission [56]. Therapeutic interventions are therefore targeted at one or two of these defects. The binding of small molecules to target proteins result in altering the conformational states of the protein beyond a certain threshold that consequentially modulate the functionalities of the protein. The design of small molecules with the aim of inducing an increase in muscle sensitization to calcium has therefore targeted the fast-skeletal-troponin complex [13]. As such, the second-generation small molecule Reldesemtiv binds to the N-terminal of the troponin C components thus slowing the rate of calcium release and therefore sensitizing the muscle to calcium. After docking Reldesemtiv into the pocket, nine binding poses with varying energy scores were generated and the pose with the highest (most negative) was selected and saved for the downstream analysis. The most negative (−7.8) with a pose RMSD of zero was selected based on reports that docking scores equal to or less than −7.0 characterizes putative from non-putative binders of protein [57] with other studies approximating that 97.7% of acknowledged binders show binding energies of −7.0 or less [57, 58] and thus filters almost 95% of non-binders [59, 60]. To ascertain the impact of the binding and to unravel the molecular determinants of this crucial therapeutic effect, we computed the root mean square deviation (RMSD), the root mean square fluctuations (RMSF), the radius of gyration (RoG) and the solvent accessibility surface area (SASA) of the C-α atoms of troponin C during the 150 ns simulation period as well as snapshot analyses of the trajectories.

### Validation Of Troponin C Via RMSD Estimations

**Fig. 2:**
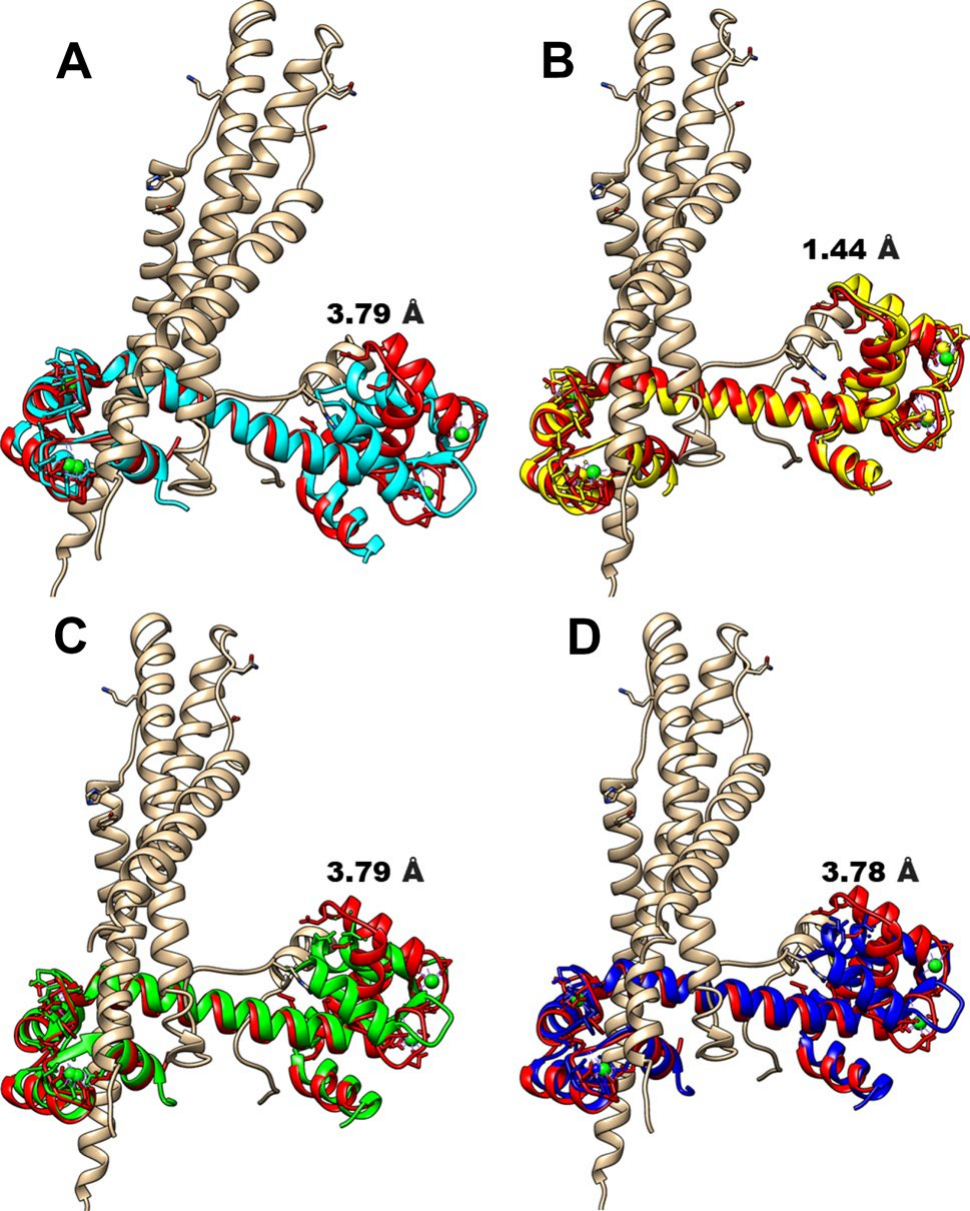
Structural comparison of the troponin C of 1YTZ used in this study to high resolution structures by estimating the root mean square deviations. **A** Shows superimposition of troponin C of 1YTZ (red) and troponin C of 5TNC (cyan) with an RMSD value of 3.79 Å. **B** Shows superimposition of troponin C of 1YTZ (red) and troponin C of 1TCF (yellow) with an RMSD value of 1.44 Å. **C** Shows superimposition of troponin C of 1YTZ (red) and troponin C of 1NCZ (green) with an RMSD value of 3.79 Å and **D** Shows superimposition of troponin C of 1YTZ (red) and troponin C of INCX (blue) with an RMSD value of 3.78 Å  (Color figure online)

Due to the current absence of a high-resolution structure of the troponin complex, validation of the structure used for these studies (1YTZ), was performed for only the troponin C component of the complex since it formed the central theme of this investigation. The root mean square deviation of this component was calculated relative to four high resolution structures with PDB IDs: 5TNC (2.0 Å) [61], 1TCF (1.90 Å) [62], 1NCZ (1.80 Å) and 1NCX (1.80 Å) [63], using the Match Maker interface of UCSF Chimera. The results generated are presented in Fig. 2 below. The RMSD was calculated for 159 residues, and they presented 3.79, 1.44, 3.79 and 3.78 Å for 5TNC, 1TCF, 1NCZ and 1NCX structures respectively. These differentials in RMSD values presented could be due to the different states of troponin C at which crystallization was performed. 1TCF which showed desirable RMSD values with the troponin C (1YTZ) was crystalized in a highly calcium saturated state while the troponin C of the complex was also crystalized in an active state thus explaining the desirable RMSD value (1.44 Å). This reposes confidence in the structure thus investigated hereafter.

### Reldesemtiv Binding Potentiates Troponin C Compacting Characterized by Reduced Exposure to Solvent Molecules

**Fig. 3:**
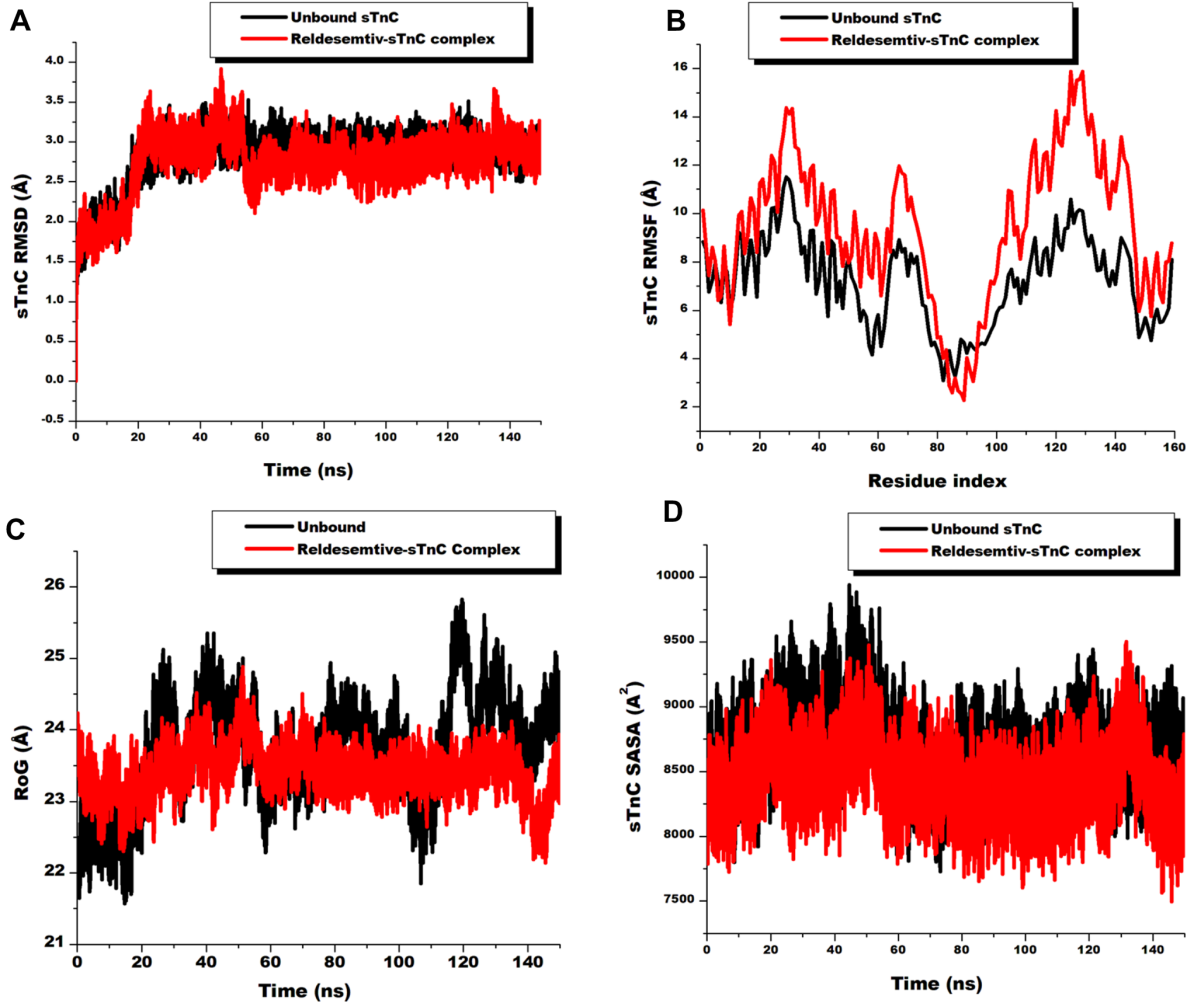
**A** Comparative RMSD plots of C-α atoms of unbound fast skeletal troponin C (black) and Reldesemtiv-sTnC complex (red) showing system stability and convergence during 150 ns simulation. **B** Comparative RMSF plots of individual residues of unbound fast skeletal troponin C (black) and Reldesemtiv-sTnC complex (red) showing increased fluctuation of Reldesemtiv complex residues. **C** Comparative RoG plots of C-α atoms of unbound fast skeletal troponin C (black) and Reldesemtiv-sTnC complex (red) depicting the compactness of the systems during 150 ns simulation. **D** Comparative SASA plots of C-α atoms of unbound fast skeletal troponin C (black) and Reldesemtiv-sTnC complex (red) showing the reduction in solvent availability surface area of the Reldesemtiv complex  (Color figure online)

We began the analysis by estimating the RMSD of the C-α atoms of the 159 residues of the troponin C component of the troponin complex to determine the stability of the protein upon Reldesemtiv binding during the simulation period. Increased atomic motions are characterized by high RMSD values and vice versa. RMSD computation revealed Redesemtiv binding could stabilize troponin C as shown in the graph presented in Fig. 3. As observed, the Reldesemtiv-troponin C complex showed average RMSD value of 2.73 ± 0.37 Å compared to the unbound troponin C which presented 2.82 ± 0.33 Å. These values also express confidence in the simulation process suggesting the systems converged during the 150 ns simulation since they fell below 3.0 Å. The RMSF estimation which is informative on the flexibility of the residues, revealed Reldesemtiv induced an increase in troponin C residual fluctuation compared to the unbound form. High RMSF values indicates a more fluctuating movement of the individual residues which collectively impact the global protein’s fluctuation. The Reldesemtiv-troponin C complex and unbound forms presented average RMSF values of 9.42 ± 3.02 Å and 7.22 ± 1.85 Å respectively as presented in Fig. 3. We further determined the impact of Reldesemtiv on the compactness of troponin C through the RoG metric. With this metric, increased values correspond to less compactness of the computed residues whiles decreased values indicates high compactness [64]. As observed from the graph presented in Fig. 3, Reldesemtiv-troponin C complex presented reduced average RoG value (23.41 ± 0.32 Å) compared to the unbound troponin C protein (23.77 ± 0.75 Å) suggesting that Reldesemtiv has the propensity of inducing high compactness of the troponin C protein. These results were corroborated by the SASA metric wherein the surface area of troponin C accessible to the solvent environment was reduced in Reldesemtiv-troponin C complex. The unbound and the complex presented average SASA values of 8684.63 ± 275.87 A^2^ and 8430.20 ± 248.30 A^2^ respectively. The SASA results further suggests the binding of Reldesemtiv could induce reorientation of residues hitherto exposed, to shift towards the hydrophobic core of the protein further reenforcing the stiffness or compactness of troponin C. In general, Reldesemtiv binding possibly induced the thickening (compacting) of troponin C in a fluctuating state. This conformational changes could lead to prolonged holding on to calcium ions and contraction of the fast skeletal muscle which could favor the slow rate of calcium release as reported in other studies [13, 20, 65].

### Reldesemtiv Complexing Detains Physiological Cations

**Fig. 4:**
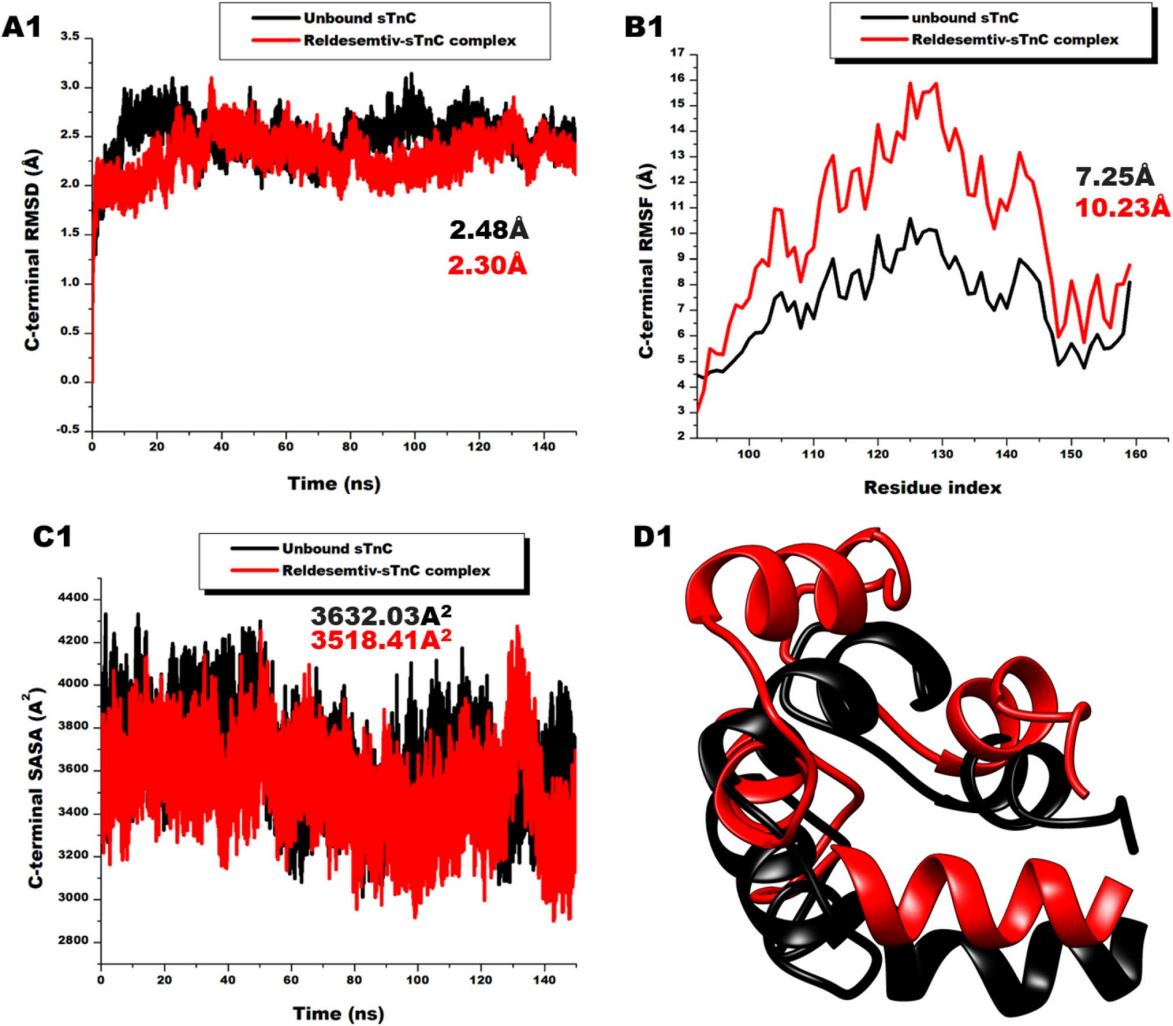

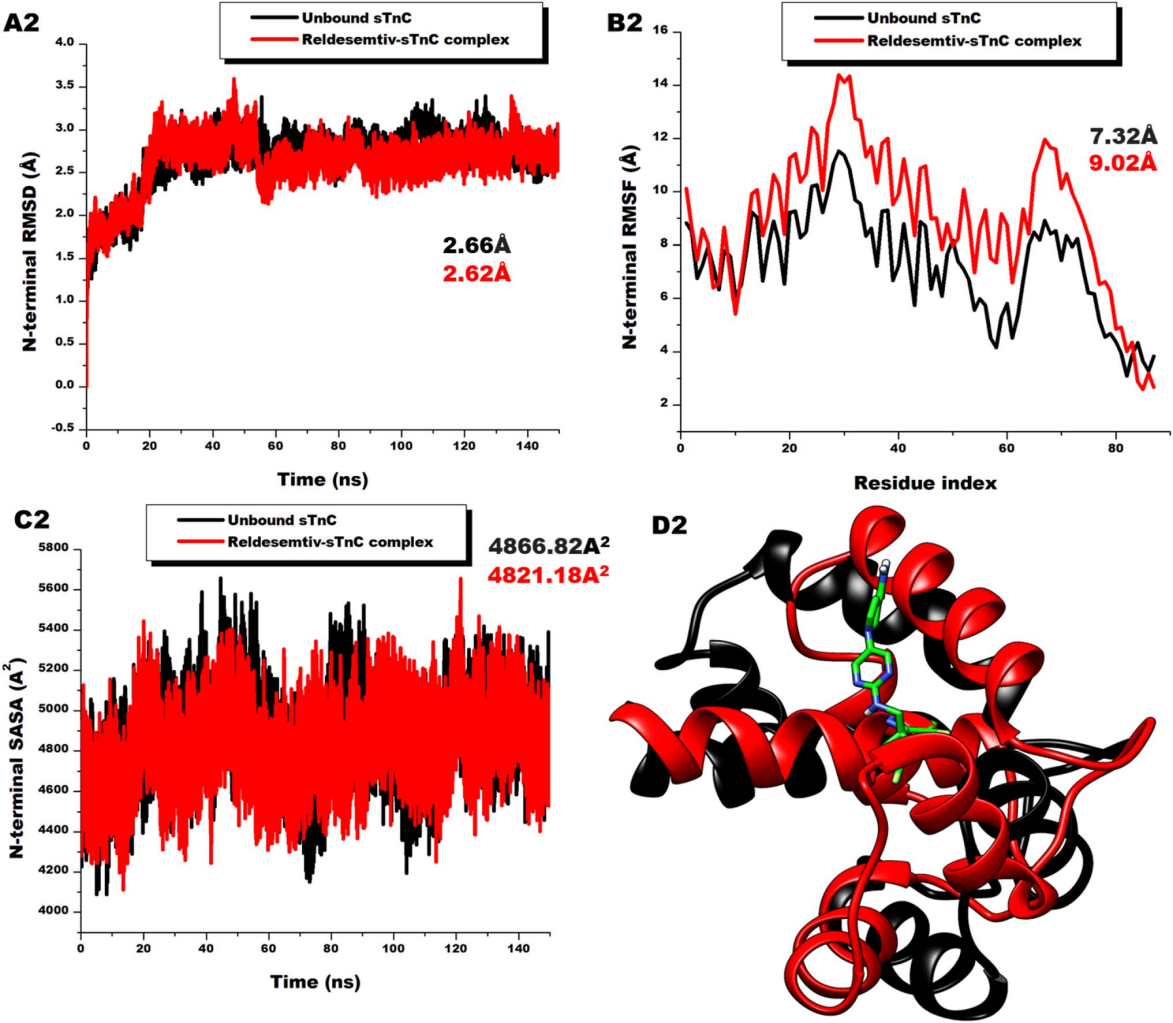
**A** (A1) Comparative RMSD plots of C-α atoms of unbound C-terminal of fast skeletal troponin C (black) and the C-terminal of Reldesemtiv-sTnC complex (red) showing system stability during 150 ns simulation (B1) Comparative RMSF plots of individual residues of the C-terminal of the unbound fast skeletal troponin C (black) and C-terminal of Reldesemtiv-sTnC complex (red) showing increased fluctuation of Reldesemtiv complex residues (C1) Comparative SASA plots of C-α atoms of the C-terminal of the unbound fast skeletal troponin C (black) and C-terminal of Reldesemtiv-sTnC complex (red) showing the reduction in solvent availability surface area of the Reldesemtiv complex terminal. (D1) Superimposition of the C-terminals of the of the unbound sTnC and the Reldesemtiv complex showing terminal flexibility. **B** Comparative RMSD plots of C-α atoms of unbound N-terminal of fast skeletal troponin C (black) and the N-terminal of Reldesemtiv-sTnC complex (red) showing system stability during 150 ns simulation (B2) Comparative RMSF plots of individual residues of the N-terminal of the unbound fast skeletal troponin C (black) and N-terminal of Reldesemtiv-sTnC complex (red) showing increased fluctuation of Reldesemtiv complex residues (C2) Comparative SASA plots of C-α atoms of the N-terminal of the unbound fast skeletal troponin C (black) and N-terminal of Reldesemtiv-sTnC complex (red) showing the reduction in solvent availability surface area of the Reldesemtiv complex terminal. (D2) Superimposition of the N-terminals of the of the unbound sTnC and the Reldesemtiv complex showing terminal flexibility. Docked in the N-terminal is Reldesemtiv (green)  (Color figure online)

After establishing the impact of Reldesemtiv on the entire troponin C, we sought to probe the differential conformational changes that may characterize the N-sTnC, and C-sTnC terminals. These terminals are linked by a short linker comprising residues 88–91[66]. Interestingly, analyses of these terminals reveal Reldesemtiv had more impact on the distant C-terminal than the N-terminal where it binds. As depicted in Fig. 4A and B, the C-terminal presented average RMSD and RMSF values of 2.30 ± 0.30 and 10.23 ± 3.10 Å respectively whiles the N-terminal showed average RMSD and RMSF values of 2.62 ± 0.31 and 9.02 ± 2.60 Å respectively. These suggest the C-terminal is more stable with residual fluctuations than the N-terminal. SASA values also showed the C-terminal is more compact (3518.41 ± 186.18 A^2^) than the N-terminal (4821.18 ± 173.11 A^2^). These differentials in N-terminal and C-terminal could also emanate from their differences in sizes. The N-terminal consist of the EF hands I and II which are reported to have low affinity for calcium ions but are highly selective to it whiles the C-terminal comprises the EF hands III and IV which are always occupied by physiological cations such as Mg^2+^ and Ca^2+^ [67, 68]. The heightened dense and compacting effect of Reldesemtiv to C-terminal could be an additional determining feature in holding on to the physiological ions. The impact of Reldesemtiv on the C-terminal could therefore be crucial in eliciting its therapeutic effect.

Summarily, Reldesemtiv’s therapeutic prowess could be hinged on its ability to tighten the C-terminal in addition to its binding (N) terminal.

### Molecular Interactions Underscoring the Therapeutic Potentials of Reldesemtiv

After establishing the conformational changes that characterize Redesemtiv binding to troponin C, we probed further to determine at the molecular and atomistic level the interactions that conditioned the conformational changes. To achieve this, snapshots generated over the simulation process were visualized and analyzed. The analyses revealed Reldesemtiv complexing is underscored by varying interactions involving hydrogen bonds, van der Waals, pi-alkyl, pi-cation, pi-sigma and pi-sulfur interactions.

**Fig. 5:**
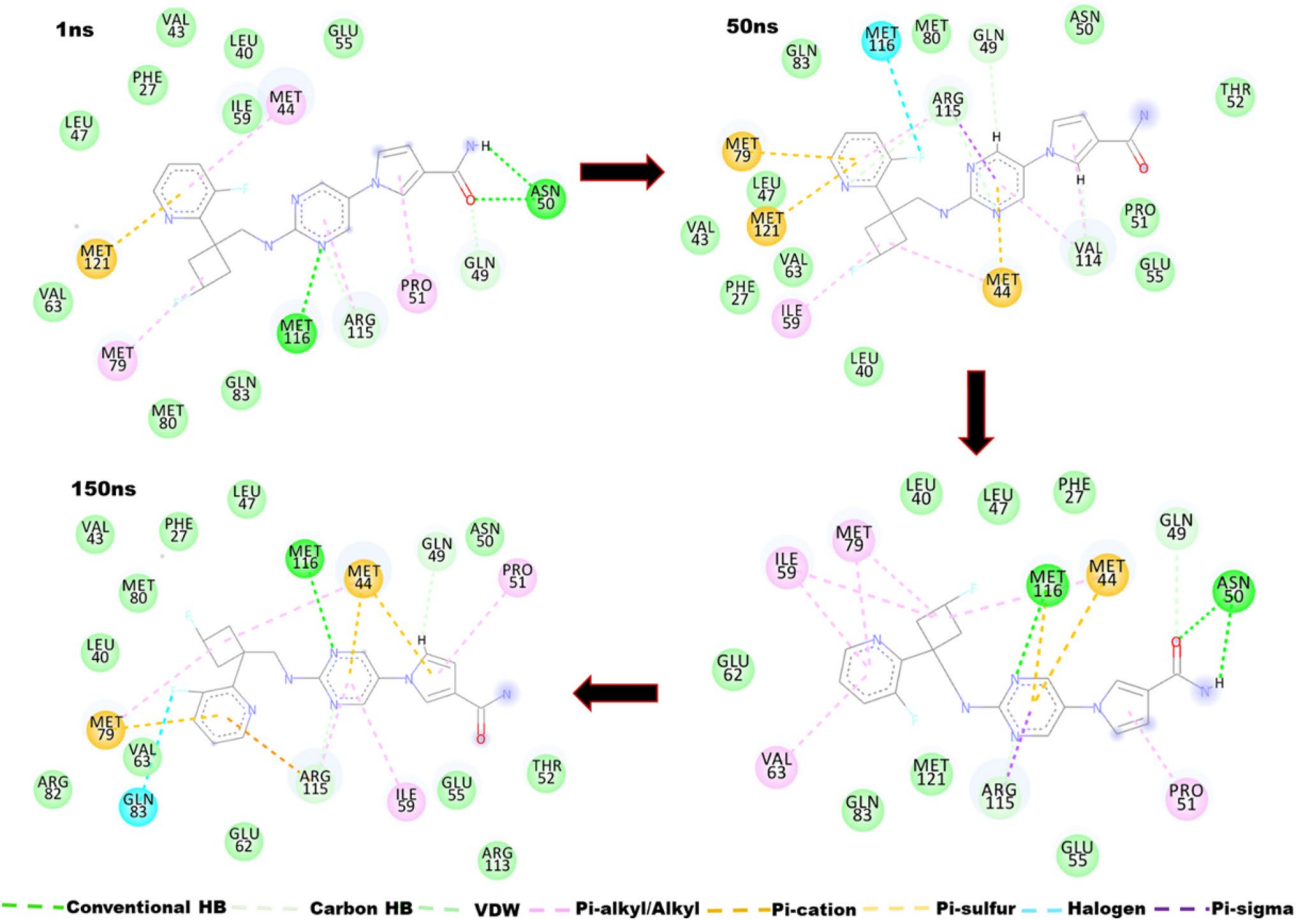
Interaction trajectory of Reldesemtiv with the N-terminal and troponin I switch residues over 150 ns simulation. Snapshots at 1, 50, 100 and 150 ns shows Reldesemtiv interacts with the residues through varying interactions involving conventional and carbon hydrogen interactions, pi-alky, pi-pi, halogen, pi-sulfur and van der Waals interactions

The interactions between Reldesemtiv and the binding site residues are displayed in Fig. 5. At 1 ns, it was observed that the sp2 hybridized O1 and H10 atoms of Reldesemtiv interacted with the H and sp2 hybridized OD1 atoms of Asn50 respectively. This interaction was again observed at 100 ns. The H atom of Met116 of sTnI also formed a hydrogen bond with the sp2 hybridized N3 atom of Reldesemtiv at 1 and 100 ns while the sp2 hybridized O atom interacted via halogen interaction with the sp3 hybridized F1 atom of Reldesemtiv. Met116 as well formed a pi-sulfur interaction with the ligand. Gln49, through the HA atom interacted via carbon hydrogen with the sp2 hybridized O1 atom of Reldesemtiv which was observed at 1 and 100 ns. As the ligand and the protein assume conformational changes during the period of simulation, new interactions were observed. At 50 ns, the OE1 sp2 hybridized atom of Gln49 formed a carbon hydrogen bond with the HC atom of Reldesemtiv. Arg115 of the sTnI of the troponin complex through HD3 and HD2 atoms interacted via carbon hydrogen bonds with the sp3 hybridized N3 atom of the ligand which was again observed at 100 ns. Again, Arg115 was observe to form alkyl and pi-sigma bonds with Reldesemtiv at 50 ns. The sTnI residue Val114 equally formed carbon hydrogen bond via the sp2 hybridized O atom with the HC14 atom of Reldesemtiv at 50 ns and a pi-alkyl bond. Other residues including Met44, Pro51, Ile59, Val63, Met79, and Met121 form various interaction types as depicted in Fig. 5.

### Free Binding Energy Calculations

The MM/PBSA method is widely employed to estimate the binding free energy of a small molecule at the atomic level [69]. This method was therefore used to estimate the total binding energy contributions of Reldesemtiv to the fast skeletal troponin complex as shown Table 1. These estimated energies offer evidence at the molecular level and could therefore lay the foundation for the development of small molecules targeting fast skeletal troponin complex with enhanced ligand binding properties. As observed, the total free binding energy, ${\mathrm{\Delta G}}_{\mathrm{bind}}$, of the reldesemtiv-fast skeletal troponin complex was −41.30 ± 3.68 kcal/mol. Interaction forces such as van der Waals (−48.95 ± 3.69 kcal/mol) and electrostatic (−25.15 ± 7.90 kcal/mol) contributed substantially to the free total binding energy of Reldesemtiv complexing. These energies suggest the complexing of Reldesemtiv to fast skeletal troponin complex is spontaneous with minimal kinetic reactions. It was also observed that the gas phase term favored the complexing whiles the solvent term opposed the complexing.

**Table 1:** Energy Profile of reldesemtiv in complex with fast skeletal troponin complex

System		Energy components (kcal/mol)				
		${\mathrm{\Delta E}}_{\mathrm{vdw}}$	${\mathrm{\Delta E}}_{\mathrm{ele}}$	${\mathrm{\Delta G}}_{\mathrm{gas}}$	${\mathrm{\Delta G}}_{\mathrm{sol}}$	${\mathrm{\Delta G}}_{\mathrm{bind}}$
Reldesemtiv		−48.95 ± 3.69	−25.15 ± 7.90	−74.11 ± 6.63	32.81 ± 7.10	−41.30 ± 3.68

ΔE_ele_ = electrostatic energy; ΔE_vdW_ = van der Waals energy; ΔG_bind_ = total binding free energy; ΔG_sol_ = solvation free energy; ΔG = gas phase free energy

### Per Residue Energy Decomposition Analysis

**Fig. 6:**
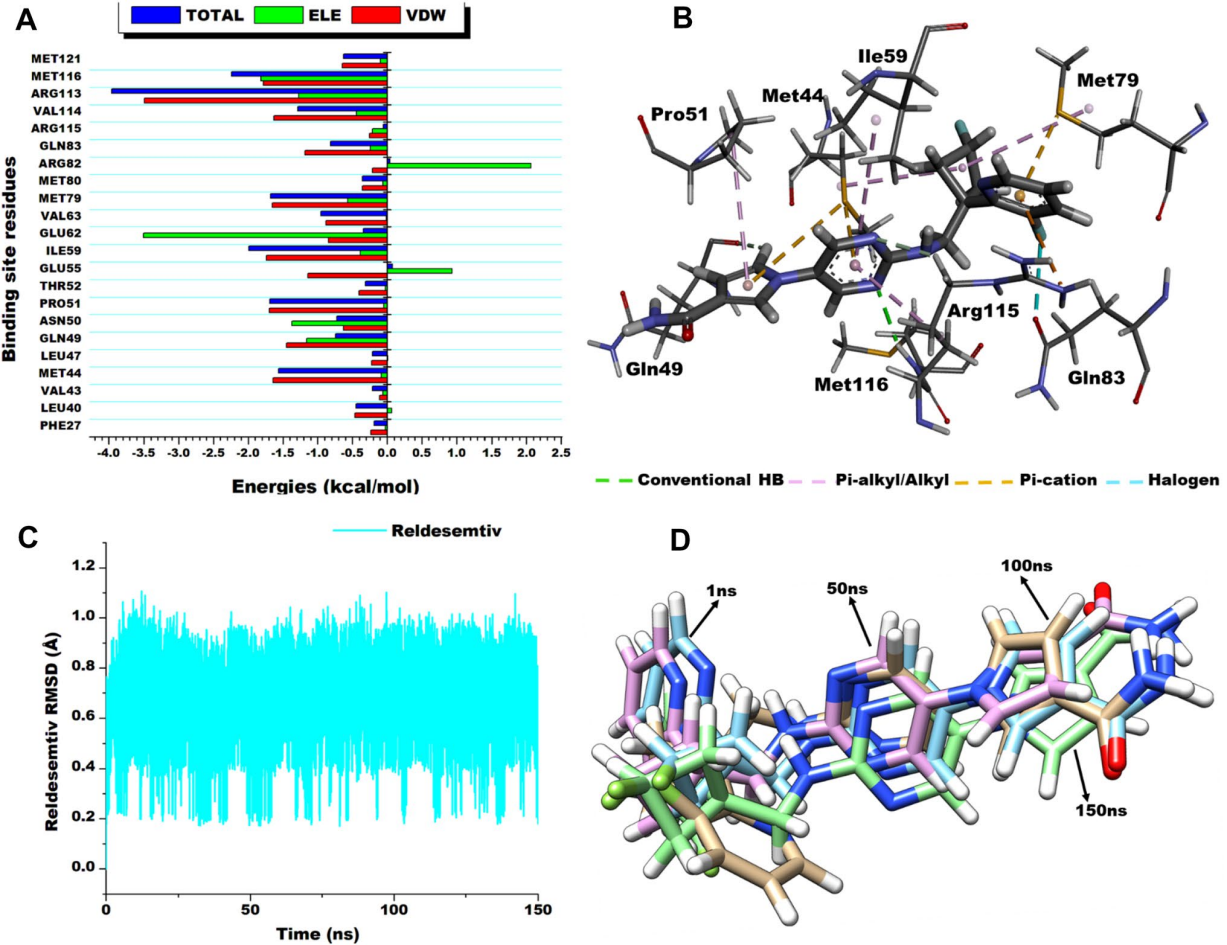
**A** Per-residue energy plots of binding sites residues towards Reldesemtiv complexing with fast skeletal troponin complex. **B** The 3D representation of intermolecular interactions exhibited by Reldesemtiv. **C** shows the plot of the RMSD of Reldesemtiv over the 150 ns simulation, graph depicts general stability within the binding pocket and **D** Superimposition of Reldesemtiv at 1, 50, 100 and 150 ns

The total free binding energy was further decomposed into individual residue contributions using the MM/PBSA method. This enabled us to determine the binding site residues interaction contributions relative to the binding free energies (van der Waals and electrostatic) of Reldesemtiv to fast skeletal troponin complex and their overall impact on the total free binding energy (${\mathrm{\Delta G}}_{\mathrm{bind}}$). The decomposed energy contributions are presented in a graph format and shown in Fig. 6. Intermolecular interactions between the binding site residues and Reldesemtiv is very crucial in its binding and stabilization within the binding pocket of fast skeletal troponin complex. As observed in Fig. 6, residues that contribute the most energy towards the complexing of Reldesemtiv included, Arg113 (−3.96 kcal/mol), Met116 (−2.23 kcal/mol), Ile59 (−1.99 kcal/mol), Pro50 (−1.69 kcal/mol), Met44 (−1.56 kcal/mol), Val114 (−1.28 kcal/mol), Val63 (−0.96 kcal/mol), Gln83 (−0.82 kcal/mol), Gln49 (−0.75 kcal/mol), Asn50 (−0.73 kcal/mol), and Met121 (−0.63 kcal/mol). Interestingly, the residues of the sTnI which interacted with Reldesemtiv are among the highest energy contributing residues (Arg113, Met116, Val114 and Met121). This indicates that the interaction of a small molecules with sTnI is very crucial in achieving its expected therapeutic effect. The effect of these interactions on the stability of Reldesemtiv within the binding pocket over the simulation period was estimated (Fig. 6D). This was determined by computing the RMSD of the ligand which presented an average figure of 0.64 ± 0.13 Å suggesting Reldesemtiv stabilized within the pocket.

## Conclusion

Direct activation of skeletal muscle has become a therapeutic intervention to improve the physical performance in patients with compromised muscle function leading to the discovery of Reldesemtiv as a second-generation drug. In our attempt to unravel the atomistic and molecular sights into the therapeutic activity of Reldesemtiv on the fast skeletal troponin complex, we docked the small molecule into the binding pocket of the troponin C subunit of the complex and then subjected the complex system to 150 ns MD simulation. Trajectories generated from the simulation period were analyzed to present relevant molecular and atomistic insights. C-a atoms analyses of the troponin C subunit of the complex revealed the binding of Reldesemtiv could have induced stability of the troponin C complex in a dense and compact state with reduced solvent accessibility surface area. Further analyses suggest Reldesemtiv induced more conformational changes on the distant C-terminal than the N-terminal where it binds taking into account the residues scale difference. These changes could result in sensitizing troponin C to calcium ions with their consequent slow release from the EF-hands. Probing further revealed the changes were conditioned by varying interactions at the binding site involving conventional and carbon hydrogen bonds, pi-pi and alkyl interactions, pi-sulfur and halogen interactions. Binding energy analyses also revealed van der Waals (−48.95 ± 3.69) and electrostatic forces (−25.15 ± 7.90) contributed significantly to the total free binding energy of Reldesemtiv (−41.30 ± 3.68). The sTnI switch region residues, Arg113 (−3.96 kcal/mol), Met116 (−2.23 kcal/mol), Val114 (−1.28 kcal/mol) and Met121 (−0.63 kcal/mol) were among the highest energy contributing residues towards underscoring their importance towards the binding and stability of Reldesemtiv. These findings therefore provide insights into the binding and impacts of Reldesemtiv on the troponin complex and would therefore be useful in the design of small molecule activators with high specificity and potency.
